# The resonant interaction between anions or vacancies in ZnON semiconductors and their effects on thin film device properties

**DOI:** 10.1038/s41598-017-02336-5

**Published:** 2017-05-18

**Authors:** Jozeph Park, Hyun-Jun Jeong, Hyun-Mo Lee, Ho-Hyun Nahm, Jin-Seong Park

**Affiliations:** 1 Department of Materials Science and Engineering, KAIST, Daejeon, 34141 Republic of Korea; 2 Department of Materials Science and Engineering, Hanyang University, Seoul, 04763 Republic of Korea; 3 Center for Correlated Electron Systems, Institute for Basic Science (IBS), Seoul, 08826 Republic of Korea; 4 Department of Physics and Astronomy, Seoul National University (SNU), Seoul, 08826 Republic of Korea; 5 R&D Center, Samsung Display, Yongin, 17113 Republic of Korea; 6 Graduate School of Nanoscience and Technology, Korea Advanced Institute of Science and Technology, Daejeon, Republic of Korea

## Abstract

Zinc oxynitride (ZnON) semiconductors are suitable for high performance thin-film transistors (TFTs) with excellent device stability under negative bias illumination stress (NBIS). The present work provides a first approach on the optimization of electrical performance and stability of the TFTs via studying the resonant interaction between anions or vacancies in ZnON. It is found that the incorporation of nitrogen increases the concentration of nitrogen vacancies (V_N_^+^s), which generate larger concentrations of free electrons with increased mobility. However, a critical amount of nitrogen exists, above which electrically inactive divacancy (V_N_-V_N_)^0^ forms, thus reducing the number of carriers and their mobility. The presence of nitrogen anions also reduces the relative content of oxygen anions, therefore diminishing the probability of forming O-O dimers (peroxides). The latter is well known to accelerate device degradation under NBIS. Calculations indicate that a balance between device performance and NBIS stability may be achieved by optimizing the nitrogen to oxygen anion ratio. Experimental results confirm that the degradation of the TFTs with respect to NBIS becomes less severe as the nitrogen content in the film increases, while the device performance reaches an intermediate peak, with field effect mobility exceeding 50 cm^2^/Vs.

## Introduction

Amorphous oxide semiconductors (AOSs) such as In-Ga-Zn-O (IGZO) with high carrier mobility are good alternatives to their amorphous silicon counterparts for the fabrication of high performance thin-film transistors (TFTs)^1^, especially in the field of flexible electronics or flat panel displays. However, the trade-off between electrical performance and stability^2^ under negative bias illumination stress (NBIS) is usually the main hurdle that delays the implementation of such devices into tangible products. For instance, high performance AOS TFTs that exhibit high field effect mobility undergo relatively large shifts in threshold voltage when subjected to negative gate bias in the presence of visible light. The latter condition emulates the operating environment of a switching element in an operating active matrix liquid crystal display (AMLCD) with a backlight unit, or in a transparent active matrix organic light emitting diode (AMOLED) TFT array exposed to ambient illumination.

In AOS TFTs, the field effect mobility is the principal metric of device performance, which is greatly influenced by the amount of free carriers and their effective mass in the semiconductor. The number of free electrons is determined by the concentration of shallow donor-like defects such as oxygen vacancies and/or hydrogen impurities, and the conduction band dispersion of the host material affects the carrier effective mass. On the other hand, the degradation of TFTs under NBIS is reported to originate from the metastable deep-to-shallow electronic transitions of bistable centers in the semiconductor^3–8^. The most trivial way to improve the reliability of oxide semiconductor devices is therefore to reduce or deactivate such electrical defects. For the latter purposes, several methods may be employed to decrease the defect density either during the semiconductor synthesis or in the associated device fabrication processes. Yet, such engineering procedures also suppress the formation of shallow donors that contribute to the total free carrier density, resulting in relatively inferior charge transport properties. In order to obtain a balance between performance and stability of AOS TFTs, a delicate control of chemical components or electronic defects is therefore mandatory.

Various electronic centers responsible for the degradation of oxide semiconductor devices have been suggested by different research groups, examples of which include bistable native defects and impurities such as oxygen vacancy (V_O_)^3^, peroxide (O_2_^2−^)^4^, interstitial oxygen (O_i_)^5^, undercoordinated In (In^*^)^6^, and interstitial or substitutional hydrogen (H_i_^7^ or H_O_^7, 8^). When they capture hole carriers, deep-to-shallow-like transitions occur by the following reactions: V_O_^0^ + 2 *h* ^+^ → V_O_^2+^, 2O^2−^ + 2 *h* ^+^ → O_2_^2−^, O_i_^2−^ + 2 *h* ^+^ → O_i_^0^, (In^*^-M)^2−^ + 2 *h* ^+^  → In^*^ (M: transition metal atom), H_i_^−^ + 2 *h* ^+^ → H_i_^+^, and HDX^−^ + 2 *h* ^+^ → H_O_^+^ (HDX: H-related DX center). Conversely, when electrons are captured, reverse transitions take place: V_O_^2+^ + 2*e* ^−^ *→* V_O_^0^, O_2_^2−^ + 2*e* ^−^ → 2O^2−^, O_i_^0^ + 2*e* ^−^ *→* O_i_^2−^, In^*^ + 2*e* ^−^ → (In^*^-M)^2−^, H_i_^+^ + 2*e* ^−^ *→* H_i_^−^, and H_O_^+^ + 2*e* ^−^ → HDX^−^. Many experimental and theoretical studies have been reported up to date on how to reduce the concentration of the above electronic defects in amorphous oxide semiconductors^9–15^. In particular, to understand the degradation of TFT devices under NBIS, an early V_O_ model has led researchers to focus on the passivation of the V_O_ related imperfections^9–14^, the incorporation of substitutional nitrogen in oxygen sites being one of a few effective methods^2, 13, 14^.

Recently, amorphous zinc oxynitride (ZnON) was experimentally demonstrated to be a semiconductor that can provide both high device performance and stability^2^. It was proposed that deep V_O_ defects may become electrically inactive if the valence band minimum of the host material were raised above the V_O_ levels, in the presence of abundant nitrogen anions. The resulting nitrogen-rich semiconductor would exhibit a band structure close to that of zinc nitride (Zn_3_N_2_) so that the V_O_ defects would not form carrier traps within the forbidden band gap, and thus influence the associated device reliability to a much lesser extent^16, 17^. However, the exact mechanism of V_O_ passivation by nitrogen in ZnON has not been elucidated to the best of our knowledge. Further studies are still required to clarify if nitrogen may actually replace an oxygen site^13, 14^ and one needs to verify if the V_O_ defects may be deactivated by having the valence band minimum of the semiconductor raised above the V_O_ levels^2^. From a point defect perspective, the following issues may be of interest: (i) Where is the V_O_ level located (deep or shallow) when nitrogen is incorporated into ZnO? (ii) Then, how do nitrogen vacancies (V_N_) and oxygen vacancies (V_O_) differ in terms of formation enthalpy and deep-to-shallow electronic transitions? (iii) How are the valence band tail (VBT) states (responsible for the formation of peroxides) affected? Moreover, in a nitrogen-rich environment with a high probability of finding two neighboring nitrogen anions or vacancies, which of a closely-paired N-divacancy complex (V_N_-V_N_) and a nitrogen dimer (N-N) is more likely to form? Understanding the above physical phenomena is therefore of the essence in order to successfully tailor the performance and stability of ZnON thin-film transistors.

The present work begins with a study on the structure of nitrogen-related defects in amorphous ZnON, based on first-principles density-functional-theory (DFT) calculations. The results indicate that (i) shallow single-donor V_N_ defects (with a deep-to-shallow-like V_N_^+^-V_N_^3+^ electronic transition similar to that of V_O_) form more easily than deep double-donor V_O_ because the Zn-N binding energy is smaller than that of a Zn-O bond, (ii) the highest occupied levels of the V_N_^+^ and V_O_^0^, which are anticipated to be photo-excited, are located near the VBT states above the valence band maximum (VBM), and (iii) the formation of O-O dimers (peroxides) that induce device degradation under NBIS becomes less likely as the nitrogen content increases. The presence of N-O dimers is not expected, considering the formation energetics. Also, although the creation of N-N dimers is energetically most favorable compared to O-O and N-O complexes, they are not likely to be generated within the anion concentration ranges that allow the realization of operating thin film transistors.

If the N concentration increases, the number of shallow V_N_^+^ defects that contribute free electrons also increases, thus enhancing the carrier mobility of the semiconductor^18, 19^. Yet, above a critical N content, the formation of electrically inactive (V_N_-V_N_)^0^ pairs is induced through a *resonant* interaction between two neighboring V_N_ vacancies. The divacancies have an effect of annihilating the electrically active V_N_^+^ defects, which diminishes the total carrier concentration. Therefore, a maximum amount of nitrogen anions is anticipated in ZnON, above which the electron mobility begins to decrease. Since the formation of O-O dimers is suppressed with increasing N content (making it less likely for an oxygen anion to find a neighboring oxygen anion), peak device performance with reasonable stability is expected to be achieved near this critical N concentration. To verify the theoretical predictions, TFTs incorporating amorphous ZnON active layers are fabricated and evaluated. It is found that indeed a O/N anion ratio of approximately 1.6 in the ZnON semiconductor results in the best balance between field effect mobility and device stability under negative bias illumination stress (NBIS).

## Results

### Mobility perspective: DFT study on vacancies and divacancies

Despite controversy, oxygen vacancies (V_O_s) in amorphous InGaZnO_4_ have been commonly regarded as both shallow and deep defects^20^, and nitrogen vacancies (V_N_s) in crystalline Zn_3_N_2_ as major shallow donors (V_N_^+^s)^21^. Among them, the shallow types have been considered as the main source of the high field effect mobility due to their ability to donate free electron carriers, while the deep V_O_s having a deep-to-shallow electronic transition, V_O_^0^ + 2 *h* ^+^ → V_O_^2+^, have been suggested to act as the main positive (hole) charge traps that induce device degradation under NBIS^3, 20^. Several research groups proposed that nitrogen may passivate the electrically active V_O_ defects in amorphous InGaZnO_4_^13, 14^ and ZnO^2^. Since anion vacancies in the amorphous ZnON may act as the sources of free carriers or hole traps, the energetics of V_O_ and V_N_ formation was studied. Individual oxygen and nitrogen vacancies were generated by respectively removing an oxygen and a nitrogen atom from the amorphous ZnON supercell. For the sake of statistical analysis, all possible vacant sites in the supercell were evaluated by static DFT calculations.

The formation enthalpy values of all possible V_O_ and V_N_ configurations in amorphous ZnON were calculated based on the following equations^22^: ${{\rm{\Omega }}}_{f}({V}_{a}^{q})=E({V}_{a}^{q})-{E}_{0}+{\mu }_{a}+q{E}_{F}$, where $E({V}_{a}^{q})$ is the total energy of the nonstoichiometric amorphous ZnON supercell including q-charged ${V}_{a}^{q}$ (*a* = N or O), *E*_0_ the total energy of a stoichiometric amorphous supercell, *μ*_*a*_ the chemical potential of either N or O, and *E*_*F*_ the electronic chemical potential. The chemical potentials of nitrogen and oxygen (*μ*_*N*_ and *μ*_*O*_, respectively) were evaluated under N-rich and O-rich (O-poor) conditions, respectively, with *μ*_*N*_ = E(N_2_)/2 and *μ*_*O*_ = E(O_2_)/2 (E(O_2_)/2 + Δ*μ*_*O*_(=δ)). Because Zn-N bong is relatively weak (the formation enthalpy of crystalline Zn_3_N_2_ is much smaller (−0.23 eV^23^) than that of crystalline ZnO (−3.64 eV^8^)), the variation of the nitrogen chemical potential (Δ*μ*_*N*_) is negligible with respect to that of the oxygen chemical potential (Δ*μ*_*O*_). Therefore the formation enthalpies of the V_O_ defects are relatively more susceptible to the film growth conditions (*δ* ≤ Δ*μ*_*O*_ ≤ 0: Δ*μ*_*O*_ = 0 and Δ*μ*_*O*_ = *δ* for O-rich and O-poor conditions, respectively). The average maximum value (δ) of the Δ*μ*_*O*_ is calculated to be approximately −2.58 eV, based on the following equation: $\delta =({E}_{0}^{ave}-({n}_{Zn}{\mu }_{Zn}^{bulk}+{n}_{N}{\mu }_{N}^{gas}+{n}_{O}{\mu }_{O}^{gas}))/{n}_{O}$(assuming that ${\mu }_{N}={\mu }_{N}^{gas}$ since Δ*μ*_*N*_ is negligible), where ${E}_{0}^{ave}$ is the average total energy of the amorphous stoichiometric supercells, ${\mu }_{Zn}^{bulk}$, ${\mu }_{N}^{gas}$, and ${\mu }_{O}^{gas}$ are, respectively, the total energies of bulk Zn metal, an isolated N atom in the gas phase, and an isolated O atom in the gas phase, and *n*_*Zn*_, *n*_*N*_, and *n*_*O*_ are, respectively, the numbers of Zn, N, and O atoms included in the supercells. All neutral V_O_^0^ defects and singly charged V_N_^+^ nitrogen vacancies were compared while maintaining *E*_*F*_ positioned at the CBM of amorphous ZnON.

**Figure 1:**
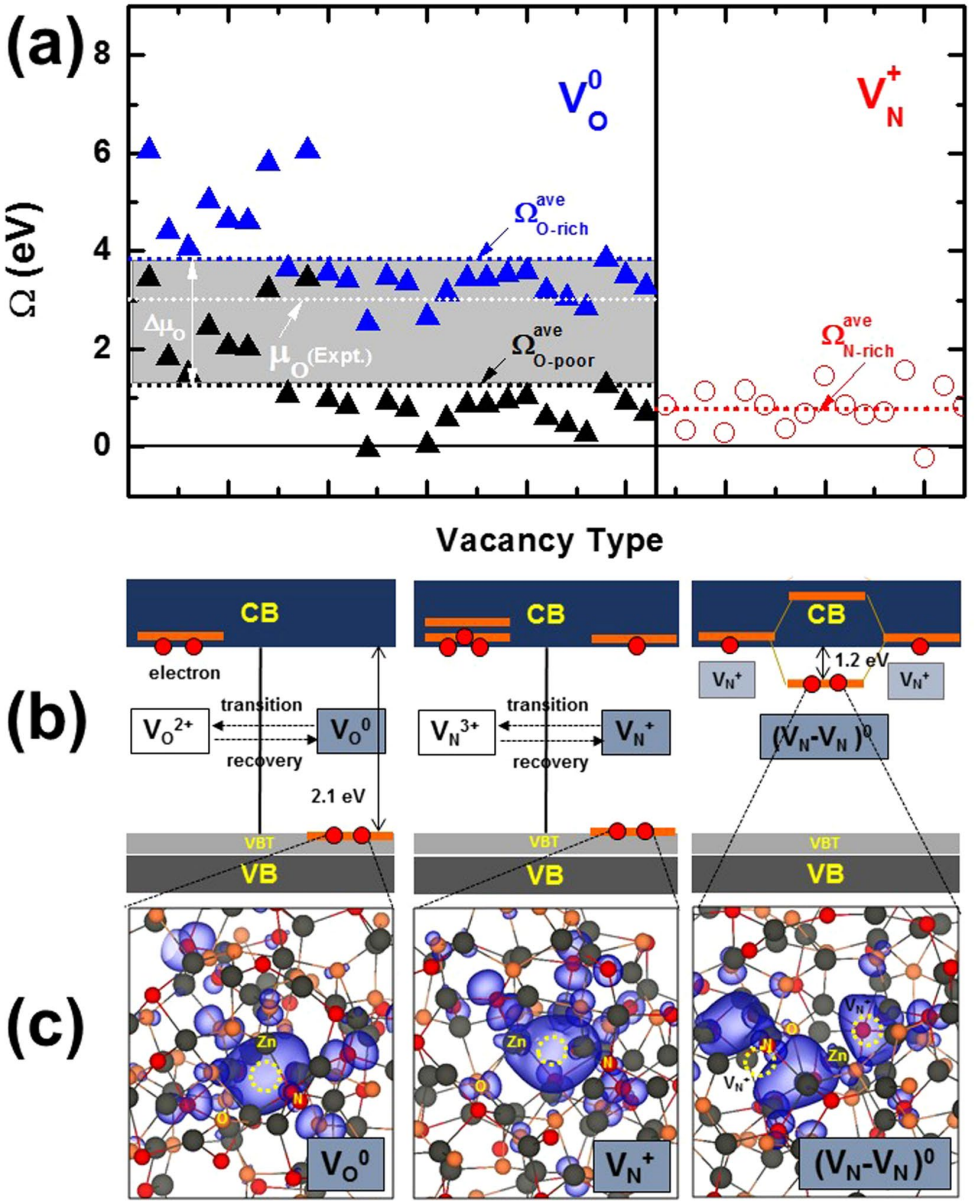
(**a**) When the Fermi level is located at the conduction band minimum (CBM), the formation enthalpy values of both shallow V_N_^+^ (red open circles) and deep V_O_^0^ defects (blue and black solid triangles for O-rich and O-poor conditions, respectively) are shown for all anion vacancies in the selected amorphous ZnON (as an example, with an O/N ratio of 1.625). Red, blue, and black short-dotted lines indicate the average formation enthalpy values of N-rich V_N_^+^, O-rich V_O_^0^, and O-poor V_O_^0^ (${{\rm{\Omega }}}_{N-rich}^{ave}$, ${{\rm{\Omega }}}_{O-rich}^{ave}$, and ${{\rm{\Omega }}}_{O-poor}^{ave}$), respectively. To compare the formation enthalpy values of the V_N_^+^ defects with those of the V_O_^0^ defects under experimental growth conditions of amorphous ZnON, the experimental oxygen chemical potential, *μ*_*O*_(*Expt*.) = *μ*_*O*_(*T*, *p*), was estimated at the growth temperature (*T* = about 300 K) and gas pressure (*p* = 5 mTorr)^24^. (**b**) The electronic defect levels of V_N_, V_O_, and nitrogen divacancy (V_N_-V_N_)^0^ are schematically depicted, respectively. VB, VBT, and CB stand for valence band, valence band tail, and conduction band, respectively. (**c**) Charge density plots of the highest occupied deep levels for V_N_^+^, V_O_^0^, and (V_N_-V_N_)^0^ are shown, respectively, with an isosurface value of 1.25 × 10^−5^ Å^−3^ (specific vacancy sites are assigned by yellow dotted circles).

Figure 1a shows the calculated formation enthalpies of the V_O_^0^ and V_N_^+^ defects in amorphous ZnON. For V_O_, opposite to the fact that deep and shallow levels may coexist in amorphous InGaZnO_4_^3, 20^, all V_O_ levels in amorphous ZnON are located deep near the VBT states. This may be understood because the resonant interaction between the somewhat perturbed dangling Zn 4 *s* orbitals around the V_O_ in amorphous ZnON is stronger than that in amorphous InGaZnO_4_ with the disorder of multi-cations. The V_O_ defect in ZnON is found to undergo a deep-to-shallow structural transition as shown in the leftmost diagram of Fig. 1b, in a way similar to the deep V_O_ levels in amorphous InGaZnO_4_, when the deep level shown in the leftmost image of Fig. 1c is occupied by two holes (see density of states (DOS) in Figure S1). The transition (E_*α*_) and recovery (E_*β*_) between V_O_^0^ and V_O_^2+^ occurs easily with negligible energy barriers. On the other hand, the V_N_ defect is found to be stable in both V_N_^+^ and V_N_^3+^ states, as shown in the middle of Fig. 1b (see DOS in Figure S2). When *E*_*F*_ is located near the CBM, the V_N_^+^ state becomes stable, acting as a shallow donor in amorphous ZnON. When two electrons are excited from the deep V_N_^+^ state shown on the middle of Fig. 1c, the structural breathing-like relaxation of the V_N_ is also accompanied with a deep-to-shallow electronic transition, similar to what is observed in the V_O_. In this case, the calculated transition and recovery barriers, E_*α*_ and E_*β*_, are also negligible.

A remarkable aspect is that the average formation enthalpy of the V_N_ defects is much lower than that of the V_O_ defects, considering the oxygen chemical potential (*μ*_*O*_(*Expt*.))^24^ under the experimental film growth conditions (temperature (*T* = about 300 K) and gas partial pressure (*p* = 5 mTorr)) to obtain amorphous ZnON samples with various O/N compositions (shown by the white shot-dot line in Fig. 1a). Furthermore, even in extremely O-poor condition, the average formation enthalpy of the V_N_ defects is smaller than that of the V_O_ defects (see Fig. 1a). Therefore as the N content increases, the number of shallow V_N_^+^ donors increases, which enhances the electron mobility in ZnON by generating more free electrons.

The possibility of obtaining divacancies by the *resonant* interaction between neighboring V_N_ defects is studied next, considering the fact that the concentration of V_N_ increases considerably with the incorporation of a large number of N anions. For this purpose, an extra V_N_ was introduced by removing a nitrogen atom in the supercell containing an initial V_N_. The binding energy of both V_N_ donors was calculated to be largely exothermic (1.29 eV) using the following equation, $(2E({V}_{N}^{+})+2{e}^{-})-({E}_{0}+E({({V}_{N}-{V}_{N})}^{0}))$. The divacancy originates from the formation of a V_N_-V_N_ bonding state induced by the resonant interaction between the two shallow V_N_ states above the CBM (as shown on the rightmost image of Fig. 1b), and thus the divacancy level is located relatively deeper at about 1.2 eV below the CBM (as shown on the right in Fig. 1c and DOS in Figure S3). When two free electrons are present, two shallow V_N_^+^ defects combine to create an electrically inactive divacancy complex: 2V_N_^+^ + 2*e* ^−^ *→* (V_N_-V_N_)^0^. The latter has an effect of annihilating the electrically active V_N_^+^ vacancies, which is anticipated to reduce the charge transport ability of the ZnON semiconductor owing to a reduction in carrier density. It is thus anticipated that as the amount of nitrogen in ZnON increases, the mobility will reach a maximum value at a certain nitrogen concentration, above which (V_N_-V_N_)^0^ pairs begin to form readily.

### Stability perspective: DFT study on anion-anion dimers

It is well-known that when two holes are captured in the valence band tail (VBT, with O-O *ppσ* ^*^ character) of amorphous InGaZnO_4_, peroxides form (2O^2−^ + 2 *h* ^+^ → O_2_^2−^) and stabilize by virtue of large lattice relaxations driven by a strong *resonant* interaction between two O-2*p* orbitals^5^. The O-O *ppσ* ^*^ level is subsequently raised above the CBM (donating two free electrons to the host). The recovery process by peroxide dissociation (O_2_^2−^ + 2*e* ^−^ *→* 2O^2−^) requires a relatively large amount of energy, since two electrons must fill-in the widely spread O-O *ppσ* ^*^ level above the CBM. The study of the electronic states on top of the valence band may be extended to ZnON semiconductors, where the effects of nitrogen become important regarding the formation of peroxide-like anion-anion dimers.

**Figure 2:**
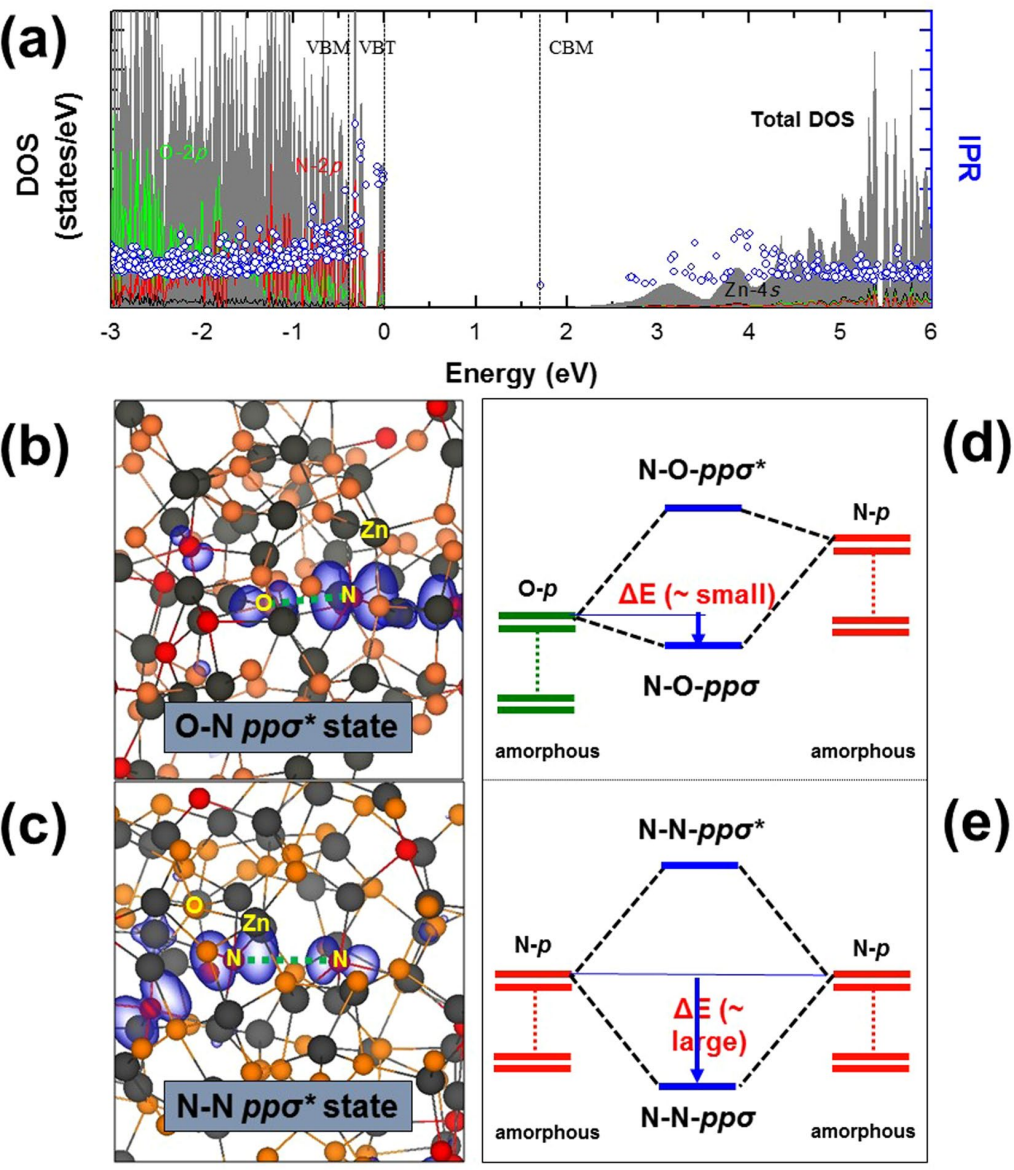
(**a**) For stoichiometric amorphous ZnON with O/N = 1.625 anion ratio as an example, the total density of states (DOS), inverse participation ratio (IPR) (indicating the degree of electronic localization), and partial DOS (PDOS) for O-2*p*, N-2*p*, and Zn-4*s* are shown. Because it is difficult to determine both the valence band maximum (VBM) and valence band tail (VBT) states of amorphous ZnON without being able to consider a crystalline ZnON structure as reference, the VBM and VBT distributions were evaluated through the analysis of the high inverse participation ratio (IPR) values near the top valance bands. For (**b**) the N-O *ppσ* ^*^ and (**c**) the N-N *ppσ* ^*^ states, charge density plots of the top valence band states in amorphous ZnON are shown with an isosurface value of 1.25 × 10^−5^ Å^−3^. The green dotted line is a bond direction along which a dimer is formed when two holes are captured at the top valence band state. Schematic formation mechanisms for the character of the top valence band states in amorphous ZnON are also shown for (**d**) the N-O *ppσ* ^*^ and (**e**) the N-N *ppσ* ^*^ states. ΔE indicates the energy gain by the anion-anion dimerization.

Figure 2a shows that since the band-gap energy of ZnON is reduced by the presence of N-2*p* orbitals, the highest valence band states in amorphous ZnON are characterized by the mixed *ppσ* ^*^ states based on O-2*p* and N-2*p* orbitals, in contrast with amorphous InGaZnO_4_ where only O-O *ppσ* ^*^ states are dominant. Figure 2b,c consist of two typical charge density plots of the VBT states in amorphous ZnON supercells, in which N-O and N-N dimers are expected to form, respectively. Three types of hole-induced anion dimers may thus be proposed in ZnON: O-O, O-N, and N-N.

First, for the O-O case, the formation of peroxides is less favorable in ZnON than in amorphous InGaZnO_4_, because the O-2*p* state is located rather deep below the VBT states, resulting in a relatively large transition energy barrier (required to have two holes occupy the corresponding *ppσ* ^*^ state). As the nitrogen concentration increases, the formation of peroxides is anticipated to be further suppressed, since the probability of an oxygen anion to find a neighboring oxygen anion decreases considerably. For N-O dimers, it may be schematically understood from the relatively small energy gain of the N-O hybridization in Fig. 2d that the N-O dimerization is not likely to take place, because the N-O *ppσ* bond energy in amorphous ZnON is weaker than that of a resonant O-O *ppσ* bond in amorphous InGaZnO_4_^4, 15^. Therefore one may suspect that the concentration of O-O (N-O) dimers will be substantially low (without any apparent influence) in ZnON semiconductors, compared to the generally reported amorphous oxide semiconductors. Finally, when two nearest N anions are present, a strong resonant interaction is induced, similar to that when peroxides form in amorphous InGaZnO_4_, as shown by a schematic description in Fig. 2e. N-N dimerization in amorphous ZnON may occur if the nearest N anions pair up. With increasing nitrogen content, a high probability of finding adjacent nitrogen anions thus promotes the formation of N-N dimers.

In order to confirm the above concepts by DFT calculations, two hole carriers were inserted in the amorphous ZnON supercells, and the possibility of anion-anion dimerization was investigated. As expected, the occurrence of peroxides and N-O dimers in proximity of the N anions is negligible in all ZnON supercells. This is because the O-O or N-O dimerizations from individual anions involve relatively high transition energy barriers (E_*α*_) and energetics, which can be overcome only when excess holes are actively present in ZnON. On the other hand, as the structural transition from 2O^2−^ to peroxide (O_2_^2−^) occurs without any apparent (or a small) energy barrier in InGaZnO_4_, the atomic and electronic structures of N-N dimers in amorphous ZnON are similar to those of peroxides in amorphous InGaZnO_4_. In the hole-injected state, the N-N structure is found to be more stable by approximately 0.96~2.89 eV (with a somewhat broad energy distribution) than the original structure in which the two N anions remain well separated. While the transition energy barriers, E_*α*_s, of the N-N formation reaction are found to be smaller than 0.1 eV, the recovery energy barriers, E_*β*_s, of the dimer dissociation reaction are relatively large (~0.83 eV). Therefore if the N concentration increases significantly, the N-N dimers may not be neglected when discussing the origins of device instability under NBIS. The above results strongly suggest that the formation of anion-anion dimers is of critical importance regarding the degradation of ZnON devices under long-time NBIS, while the fast transitions of V_N_^+^ and V_O_^0^ defects are expected to induce temporary effects only.

### Mobility and stability of ZnON-based thin-film transistors: Experimental confirmation

The actual anion contents in the ZnON films grown with different gas flow rates were measured by Auger electron spectroscopy (AES), and are listed in Table 1.

**Table 1:** Relative oxygen to nitrogen anion ratio in the ZnON films analyzed by Auger electron spectroscopy (AES), with respect to the incorporated nitrogen gas flow rate ratio [N_2_/(O_2_ + N_2_)] during growth.

N_2_/(O_2_ + N_2_) Gas Flow Rate	Anions in film (AES)		
	O (%)	N (%)	O/N
94.4	35.4	9.4	3.77
95.7	31.2	12.9	2.42
97.1	26.5	16.5	1.61
98.5	23.0	18.4	1.25

**Figure 3:**
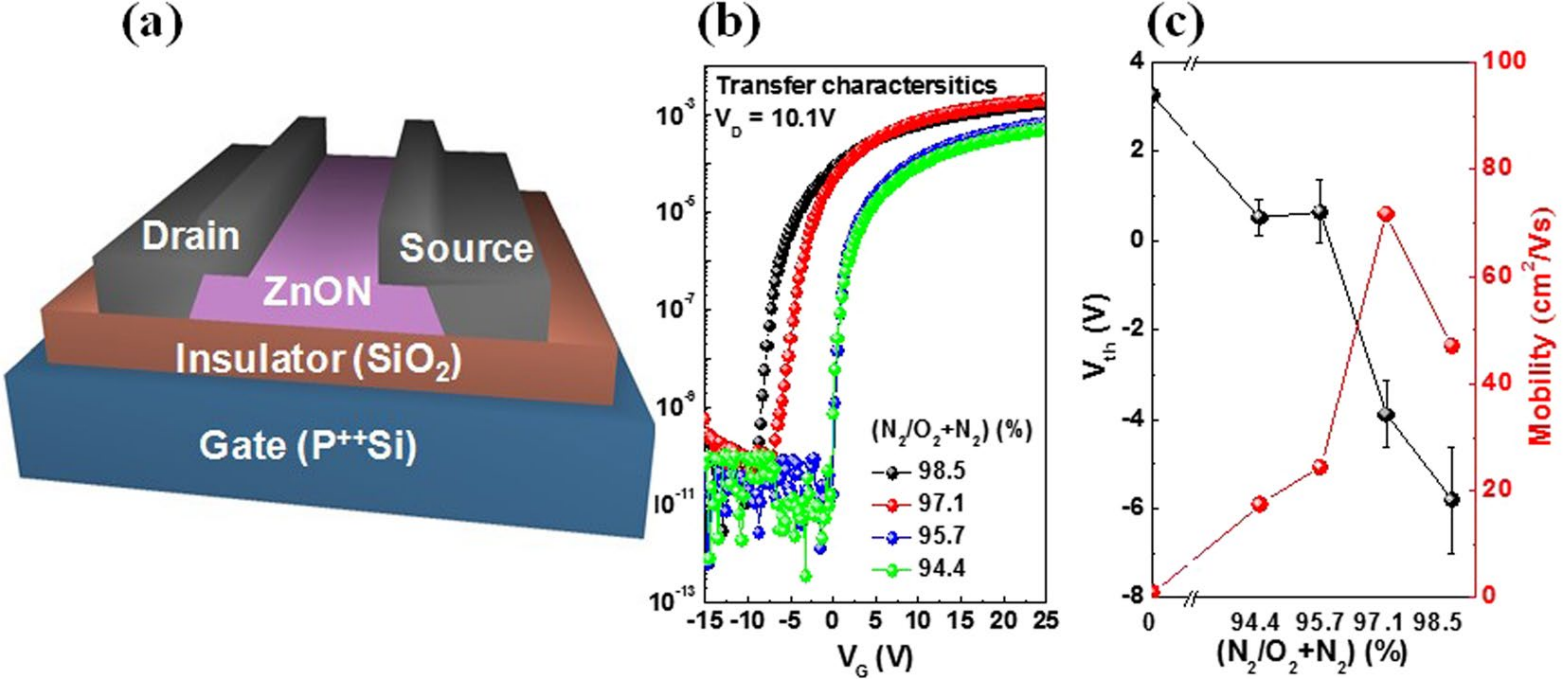
(**a**) Schematic diagram of a ZnON TFT device. (**b**) Transfer characteristics of the devices incorporating ZnON active layers grown with different N_2_/(N_2_ + O_2_) gas flow rate ratios. (**c**) Field effect mobility (*μ*_*FE*_) and threshold voltage (V_th_) with respect to the N_2_/(N_2_ + O_2_) gas flow rate ratios used during ZnON growth.

Figure 3a shows the schematic structure of a unit TFT device incorporating a ZnON active layer. Figure 3b represents the transfer characteristics that show the drain current (I_D_) with respect to the gate voltage (V_G_), as a function of the N_2_/(N_2_ + O_2_) flow rate ratio during ZnON growth. The field effect mobility (*μ*_*FE*_) and threshold voltage (V_th_) values with respect to the N_2_/(N_2_ + O_2_) flow rate ratio are plotted in Fig. 3c. Note that as the nitrogen content increases, the mobility reaches a peak value at N_2_/(N_2_ + O_2_) = 97.1%, and decreases with further nitrogen gas incorporation, just as was expected in the DFT calculations regarding the formation of (V_N_-V_N_)^0^ pairs.

**Figure 4:**
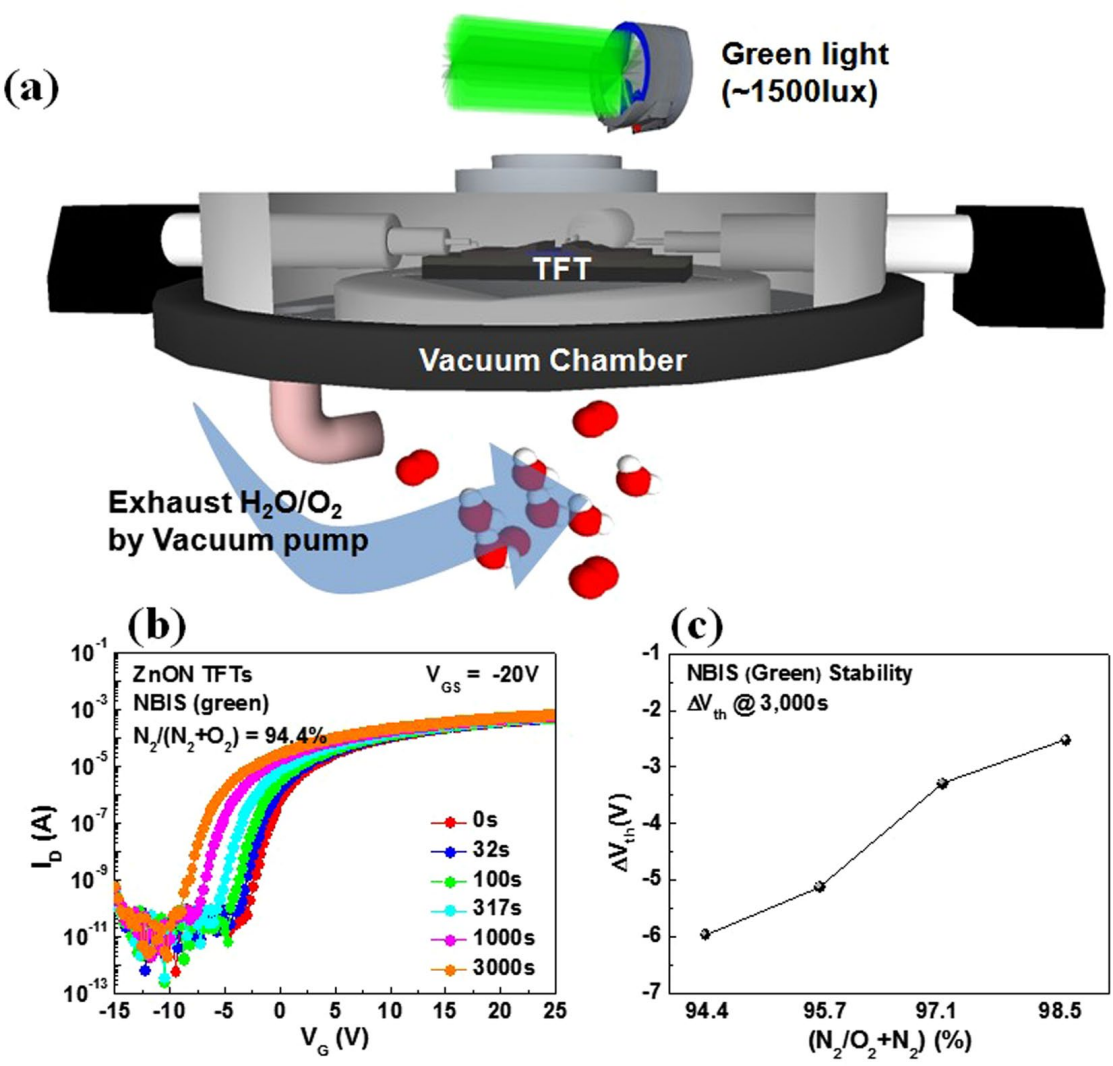
(**a**) Experimental setup for the measurement of ZnON TFT characteristics and stability, using a vacuum chamber and a green light source on top of the entire compartment. (**b**) Transfer characteristics under NBIS of a TFT based on ZnON grown with N_2_/(N_2_ + O_2_) = 94.4%. (**c**) V_th_ shift (ΔV_th_) under NBIS of the ZnON TFTs with respect to the N_2_/(O_2_ + N_2_) gas flow rate ratio used during ZnON growth.

**Figure 5:**
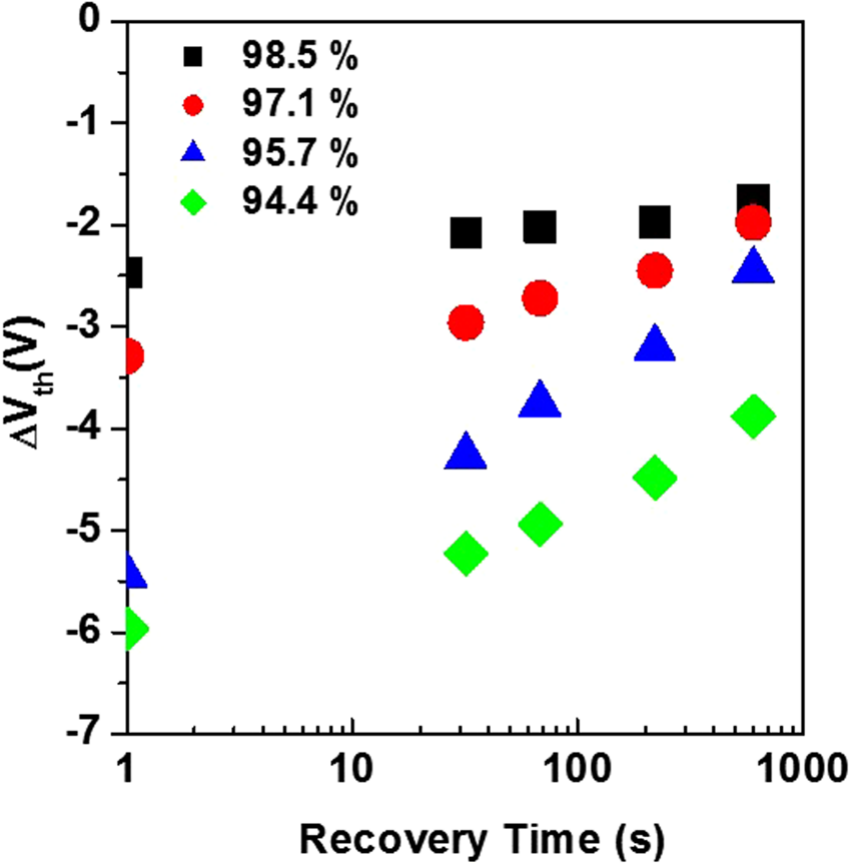
The change in ΔV_th_ values after NBIS, as the devices are left to recover with the light turned off and without any voltage bias. Note that the devices based on ZnON grown with the highest oxygen content exhibit slowest recovery, which agrees well with the theoretical predictions that O-O peroxides will result in relatively slow recovery from the photo-excited state.

Figure 4a illustrates the TFT evaluation scheme, involving a monochromatic source of green light, and an evacuated chamber in which the device is positioned in order to exclude all environmental artifacts such as moisture permeation. Figure 4b shows the time evolution of the transfer curves of a ZnON TFT fabricated with N_2_/(N_2_ + O_2_) = 94.4%, under NBIS for a total stress time of 3,000s. Figure 4c shows a plot of total V_th_ shift (ΔV_th_) of the devices under NBIS, with respect to the nitrogen gas flow rate during ZnON growth. Note that as the nitrogen incorporation increases, the amount of device degradation under NBIS is reduced. Figure 5 consists of the change in ΔV_th_ values of the devices after NBIS, as they are left to recover without any light or voltage bias. While the TFTs based on ZnON active layers grown with N_2_/(O_2_ + N_2_) gas flow rates of 98.5, 97.1 and 95.7% exhibit relatively fast recovery to ΔV_th_ values close to −2 V, the device containing the highest amount of oxygen (grown with N_2_/(O_2_ + N_2_) = 94.4%) undergo relatively slow recovery, reaching a final ΔV_th_ value of approximately −3.86 V. The ΔV_th_ values during NBIS and after the stress test are listed in Table 2.

**Table 2:** Amount of Vth shift (ΔV_th_) during NBIS for 3000 seconds, and the ΔV_th_ values after the devices are left to recover from the bias stress for 1000 seconds, with respect to the O/N ratio in the films (measured by AES).

N_2_/(O_2_ + N_2_) Gas Flow Rate (O/N ratio by AES)	ΔV_th_ (NBIS, 3000s)	ΔV_th_ after recovery
94.4 (3.77)	−5.97	−3.88
95.7 (2.42)	−5.44	−2.45
97.1 (1.61)	−3.29	−1.98
98.5 (1.25)	−2.47	−1.76

The ΔV_th_ values are all measured with reference to the initial V_th_ values before the NBIS stress.

## Discussion

The field effect mobility, *μ*_*FE*_, of amorphous ZnON-based TFTs increases as the free carrier density increases. The latter is determined by the concentration of N anions in the ZnON semiconductor. As the N_2_/(N_2_ + O_2_) flow rate ratio is increased, the total N content increases, which results in relatively low electron effective mass in the ZnON layer^2^. An important finding is the fact that the present ZnON-based TFTs exhibit peak *μ*_*FE*_ at a critical N content. Above this value, the sudden decrease of the *μ*_*FE*_ observed in the samples may be theoretically understood by either the decrease of free carriers (considering the scattering mechanisms suggested as percolation^25^, hopping^26^, or cation disorder models^27^) or another mechanism (i.e., further ionized impurity scattering well-known in general semiconductors). In this regard, the density of free electrons in the grown amorphous ZnON samples was studied as a function of N content through Hall measurements. As the N_2_/(N_2_ + O_2_) gas flow rate ratio increases in the following order: 94.4, 95.7, 97.1 and 98.5%, the corresponding films’ free carrier density values are 9.29 × 10^17^, 2.58 × 10^18^, 4.92 × 10^19^ and 1.44 × 10^19^, respectively. The sudden decrease in *μ*_*FE*_ at N_2_/(N_2_ + O_2_) = 97.1% is thus highly likely to occur from the decrease in free carrier density, which indicates that the concentration of shallow donor-like defects such as vacancies and/or hydrogen impurities must have been reduced. Hydrogen impurities are generally well known to act as effective shallow donors in various oxide semiconductors. However, in the present work the electrical properties of the ZnON layers are controlled by the N_2_/(N_2_ + O_2_) flow rate ratio during ZnON growth without intentional hydrogen incorporation, therefore vacancies should be regarded as the major source of free carriers. Here, the nonlinear relationship between mobility and carrier concentration complies well with the formerly reported transport theories such as percolation^25^, disorder scattering^27^, and extended mobility edge^28^. From the DFT calculations, the existence of a peak mobility may be concluded to originate from the reduction of V_N_^+^ donors by the formation of the electrically inactive nitrogen divacancies, (V_N_-V_N_)^0^, above a critical N content.

**Figure 6:**
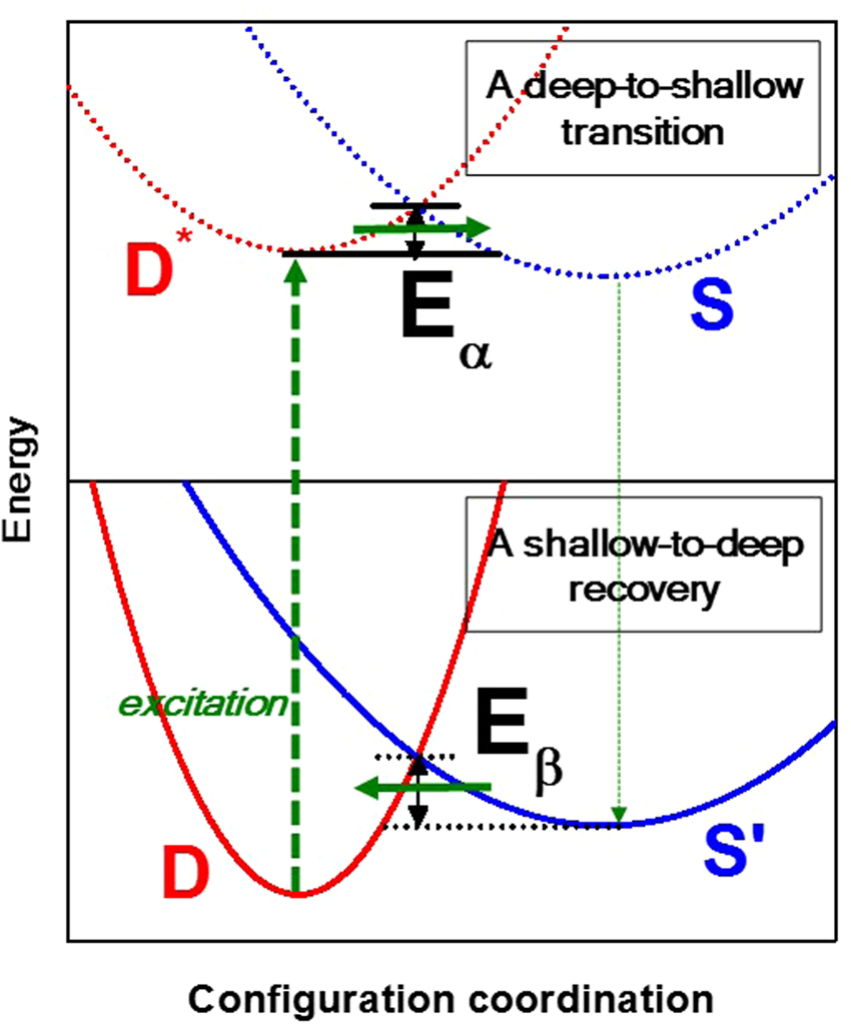
Schematic energy diagram for the hole-induced transition (top) and recovery (bottom) between deep and shallow states in bistable centers, explained in terms of configuration coordinates. D (D^*^) and S (S^’^), respectively, indicate the deep states without (with) the excitation of electron carriers [*i*.*e*. V_O_^0^, V_N_^+^, and 2O^2−^ (V_O_^0^ + 2 *h* ^+^, V_N_^+^ + 2 *h* ^+^, and 2O^2−^ + 2 *h* ^+^)] and the shallow ones without (with) free electron carriers [i.e., V_O_^2+^, V_N_^3+^, and O_2_^2−^ (V_O_^2+^ + 2*e* ^−^, V_N_^3+^ + 2*e* ^−^, and O_2_^2−^ + 2*e* ^−^)]. E_*α*_ and E_*β*_ are, respectively, the energy barriers for the deep-to-shallow transition and shallow-to-deep recovery.

Bistable centers provide a good explanation on the microscopic origins of the negative shifts in threshold voltage (V_th_) under NBIS (see Fig. 6). As mentioned in the above sections, the instability of amorphous ZnON devices with respect to NBIS is influenced by the V_O_^0^, V_N_^+^, or O-O dimers that undergo deep-to-shallow transitions. When two holes excited by light are captured at the deep levels, the transitions of V_O_^0^, V_N_^+^, or anion dimers occur instantly without any apparent energy barrier (E_α_ < 0.1 eV): V_O_^0^ + 2 *h* ^+^ → V_O_^2+^, V_N_^+^ + 2 *h* ^+^ → V_N_^3+^, or 2O^2−^ + 2 *h* ^+^ → O_2_^2−^. Then, two photo-excited free electron carriers may exist in a metastable state near the CBM in the form of V_O_^2+^ + 2*e* ^−^, V_N_^3+^ + 2*e* ^−^, or O_2_^2−^ + 2*e* ^−^ complexes. The lifetime of the metastable states is determined by the recovery energy barrier (E_*β*_). As shown in the above calculation results, the V_N_^+^ (V_O_^0^) defects require relatively short recovery times from the V_N_^3+^ (V_O_^2+^) states; i.e. V_N_^3+^ + 2*e* ^+^ → V_N_^+^ (V_O_^2+^ + 2*e* ^+^ → V_O_^0^), since E_*β*_ < 0.1 eV. On the other hand, the O-O dimers (O_2_^2− + ^2*e* ^+^ → 2O^2−^) require relatively long recovery times from the peroxide state, since E_*β*_ ~ 0.97 eV^4^. Therefore, relatively high N contents favor the stability of ZnON devices with respect to NBIS, because the formation of O-O dimers is suppressed by the presence of nitrogen anions in ZnON. This is consistent with the experimental results, in which the shifts in threshold voltage (ΔV_th_) decrease with increasing nitrogen content in ZnON during NBIS. After all stresses are released, the device based on ZnON containing the largest amount of oxygen undergoes indeed slowest recovery.

## Conclusions

In the present work, the influence of nitrogen-related defects on the electrical properties of amorphous ZnON was theoretically and experimentally examined. The *resonant* interaction between anions or anion vacancies is found to play a fundamental role in the formation of individual anion vacancies (V_N_ and V_O_), anion-anion divacancies, and anion-anion dimers. First-principles calculations indicate that the formation of shallow-donor nitrogen vacancies (V_N_^+^) is energetically favorable in comparison with that of deep-donor oxygen vacancies (V_O_^0^) when *E*_*F*_ is equal to the CBM. If the nitrogen content increases beyond a critical point, the formation of electrically inactive (V_N_-V_N_)^0^ pairs decreases the free carrier density, so that a decrease in electron mobility is observed.

Regarding the formation of anion-anion dimers, the incorporation of nitrogen modifies the valence band structure in such a way to suppress the formation of O-O dimers, which are anticipated to be the major defects that induce the degradation of ZnON TFTs under NBIS. Peroxides are less likely to form, since the probability of having neighboring O-O pairs decreases considerably as the nitrogen content increases. As a result, device stability is enhanced with increasing nitrogen concentration. From the above observations, it is reasonable to conclude that maximum performance and reasonable stability of TFTs based on amorphous ZnON semiconductors may be achieved by controlling the anion composition. Experimental results involving the fabrication of ZnON transistors indeed confirm this hypothesis, suggesting that an optimum O/N ratio of approximately 1.6 in the active layer may result in the highest field effect mobility, while the device reliability may be further improved with increasing nitrogen content.

## Methods

### First-principles calculations

The Vienna Ab-initio Simulation Package (VASP)^29^ was used for first-principles calculations. Melt-and-quench molecular dynamics (MD) simulations were performed to obtain stoichiometric amorphous ZnON supercells based on the initial configurations derived from previously-studied amorphous ZnON structures^2^, and static density-functional-theory (DFT) calculations were adopted to generate the atomic and electronic structures of the stoichiometric amorphous structures, as well as cells containing vacancies, divacancies, and anion-anion dimers. Projector augmented wave (PAW) pseudopotentials^30^ were used, along with a plane wave basis set with a kinetic energy cutoff of 400 eV and a 2 × 2 × 2 **k**-point mesh (on exception, a 1 × 1 × 1 **k**-point mesh was used for the MD simulation). While the Perdew-Burke-Ernzerhof (PBE)^31^ exchange-correlation functional was applied during the MD simulations, the generalized gradient approximation (GGA) plus *U* (GGA + *U*) functional^32^ and the hybrid functional of the Heyd-Scuseria-Ernzerhof (HSE) type^33^ with a screening parameter of 0.2 Å^−1^ were used in the static DFT calculations. A *U* value of 6.5 eV was chosen, which is in good agreement with the former DFT calculations^34^, and the HSE mixing parameter was set to describe an intermediate value, *α* = 0.355, between the mixing parameter of crystalline Zn_3_N_2_ (0.325) and ZnO (0.375)^8, 35^.

To study the effects of the O/N anion ratio in stoichiometric amorphous ZnON, three types of supercells were constructed, each including a total number of 101, 92, and 100 atoms, with O/N = 3.80, 1.625, and 1.250 (N/(N + O) = 20.83, 38.09, and 44.44 at.%), respectively. For each type, at least four samples were generated by melt-and-quench MD simulations, as illustrated in Figure S4. In case of the charged defects, the supercell size effect was corrected by considering the spurious electrostatic interaction^36^. Since the GGA + *U* calculations tend to underestimate the band gap values of amorphous ZnON, the band gap correction scheme was applied by considering the HSE hybrid calculations. The calculated band gaps of three type ZnON semiconductors with O/N = 3.80, 1.625, and 1.250 are, respectively, estimated to be about 1.38~2.53, 1.10~1.73, and 0.98~1.74 eV (see Figures S5–S7). These are in good agreement with experimentally determined optical band gaps of the stoichiometric amorphous ZnON semiconductors (1.27 eV, 1.01, and 0.92 eV, respectively). In case of the shallow V_N_ defects, the band filling correction was applied to the neutral charge state (the neutral charged V_N_^0^ is regarded as a singly charged V_N_^+^ with an electron in the conduction band minimum (CBM): V_N_^+^ + *e* ^−^).

### Materials and characterization

The fabrication of ZnON layers involved the use of direct current (DC) reactive sputtering using a zinc (Zn) metal target, onto *p* ^++^ Si/SiO_2_ (SiO_2_: 100 nm thick) substrates. The DC power was 100 W and the process pressure was kept at 5 mTorr without intentional heating of the substrates. A mixture of Ar/O_2_/N_2_ gas was used to generate the plasma, and the nitrogen gas flow rate (N_2_/(N_2_ + O_2_)) was varied from 94.4 to 98.5%. For both thin film analyses and TFT fabrication, the ZnON layer thickness was maintained between 30~35 nm. To fabricate the devices, the active islands were patterned using shadow masks. To form the source-drain electrodes, a 100 nm-thick indium tin oxide (ITO) film was sputter deposited every time, using shadow masks as well. The resulting channel width and length of the devices are 800 and 200 µm, respectively. The electrical properties of the TFTs were evaluated using a HP4155A semiconductor parameter analyzer in a vacuum ambient in order to exclude environmental effects such as moisture permeation into ZnON. For the NBIS tests, the gate voltage (V_G_) was maintained at −20 V, and the drain voltage (V_D_) was kept at +10.1 V.

## Electronic supplementary material

Supplementary Information
