# Supplementary material for: The resonant interaction between anions or vacancies in ZnON semiconductors and their effects on thin film device properties
Source: Sci Rep. 2017 May 18;7:2111. doi: 10.1038/s41598-017-02336-5 (PMC5437099; doi:10.1038/s41598-017-02336-5)
Supplement: Supplementary file 1 — Supplementary Information [file 41598_2017_2336_MOESM1_ESM.pdf]

Supplementary Information

The resonant interaction between anions or vacancies in ZnON semiconductors and their effects on thin film device properties

Jozeph Park[1,a]*, Hyun-Jun Jeong*[2,a]*, Hyun-Mo Lee*[2]*, Ho-Hyun Nahm*[3,4*]*, and Jin-Seong Park*[2*]

[1]Department of Materials Science and Engineering, KAIST, Daejeon 34141, Republic of Korea

[2]Department of Materials Science and Engineering, Hanyang University, Seoul 04763, Republic of Korea

[3]Center for Correlated Electron Systems, Institute for Basic Science (IBS), Seoul 08826, Republic of Korea

[4]Department of Physics and Astronomy, Seoul National University (SNU), Seoul 08826, Republic of Korea

[*]Corresponding Authors: jsparklime@hanyang.ac.kr & hohyunnahm@snu.ac.kr

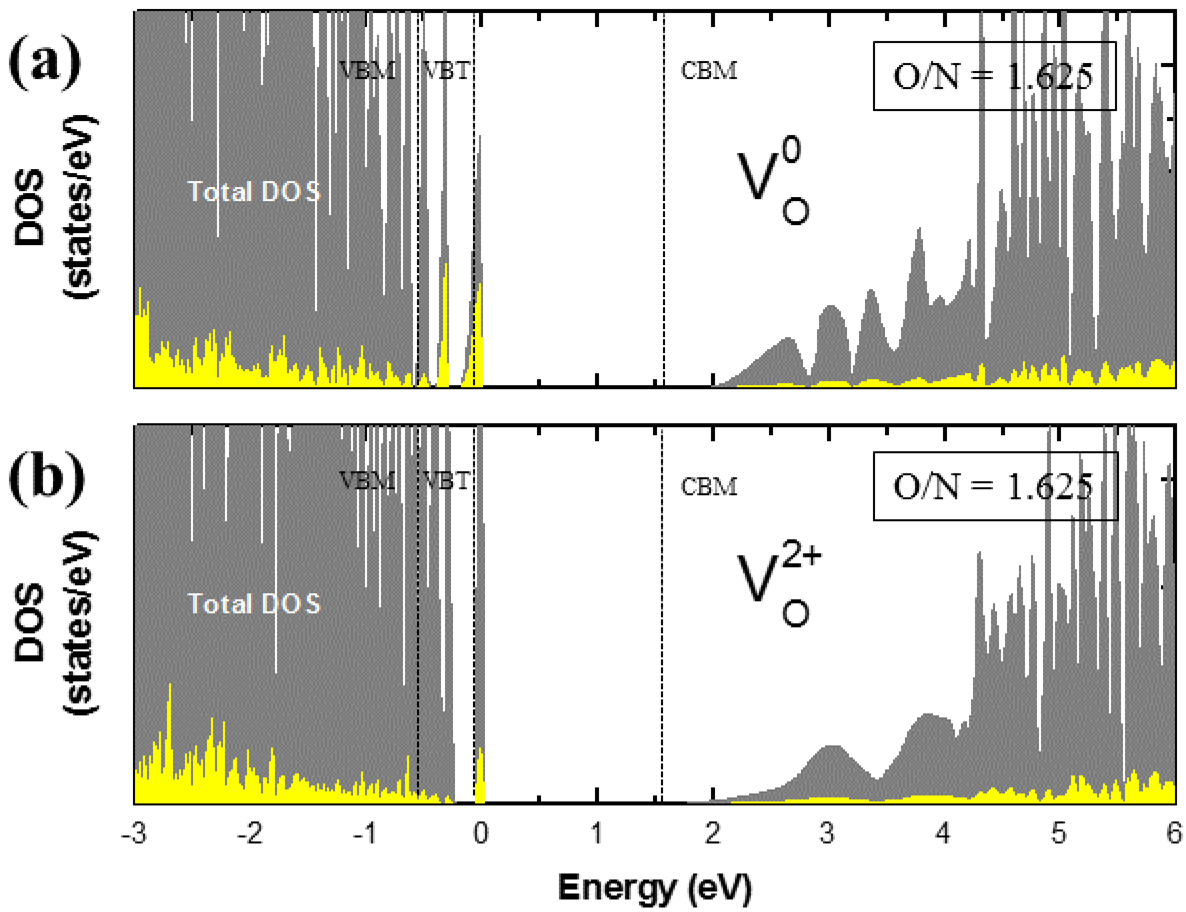

Figure S1. For V_O of an amorphous ZnON sample with O/N=1.625 anion ratio, the total DOS (gray-color) and the partial DOS (PDOS) (yellow-color) for Zn atoms near V_O are shown in (a) the (0)-charge state and (b) the (2+)-charge state, from first-principles Heyd-Scuseria-Ernzerhof (HSE) calculations.

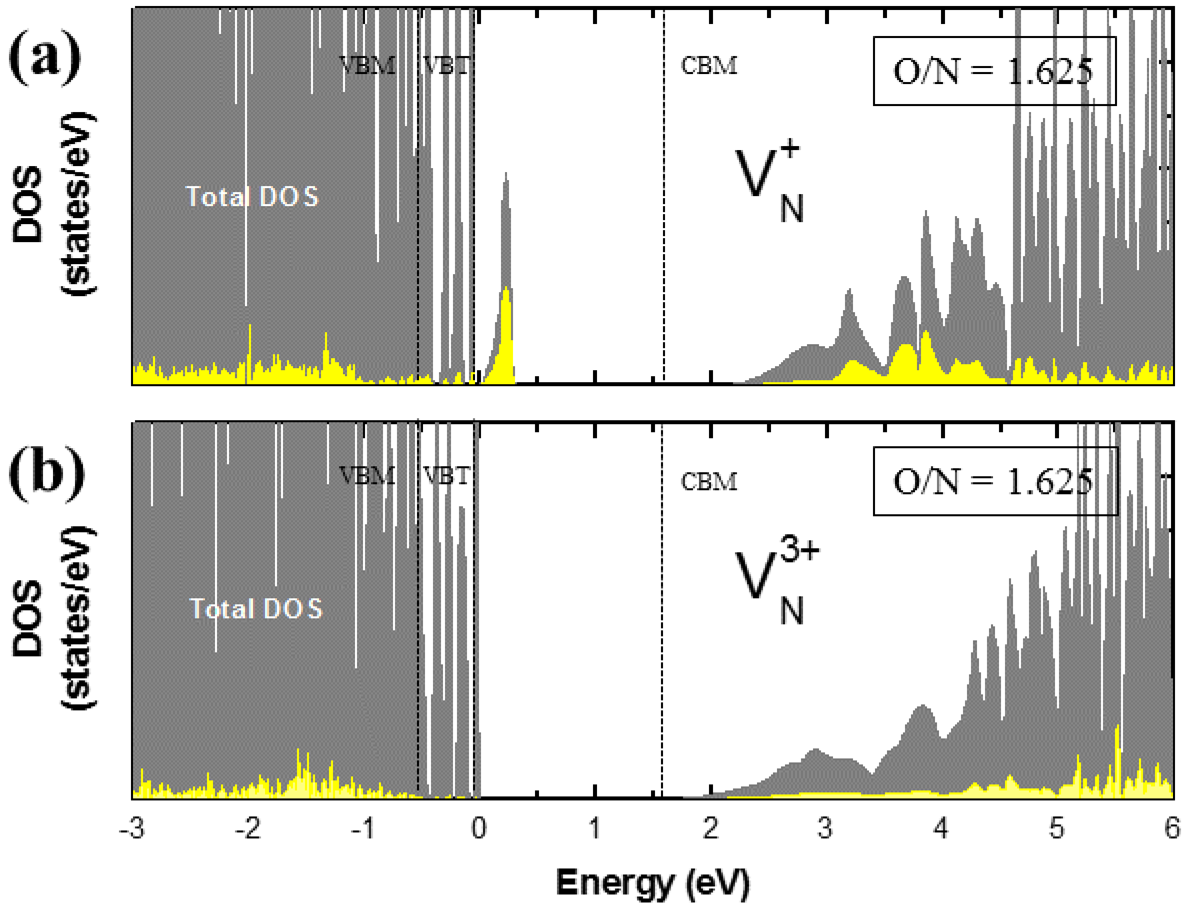

Figure S2. For V_N of an amorphous ZnON sample with O/N=1.625 anion ratio, the total DOS (gray-color) and PDOS (yellow-color) for Zn atoms near V_N are shown in (a) the (+)-charge state and (b) the (3+)-charge state, from first-principles HSE calculations.

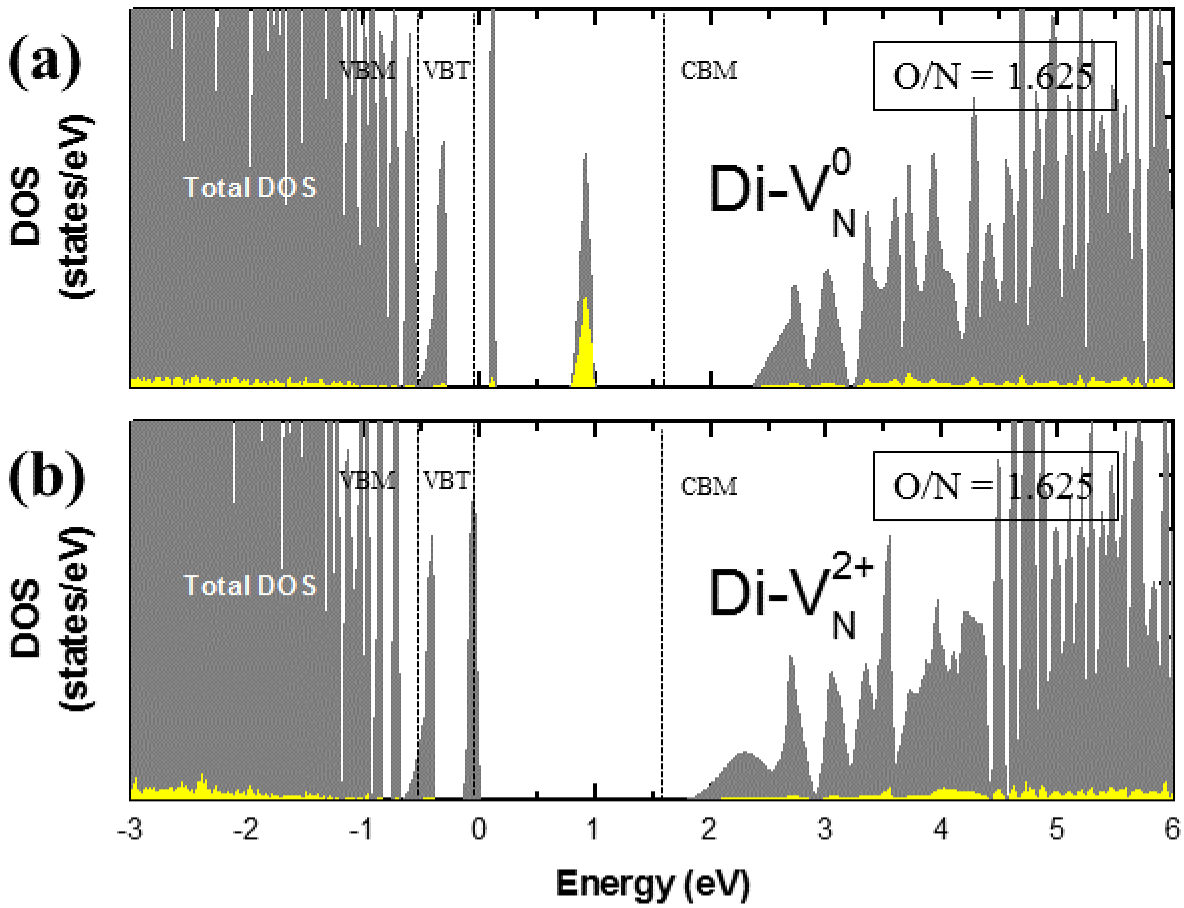

Figure S3. For divacancy of an amorphous ZnON sample with O/N=1.625 anion ratio, the total DOS (gray-color) and PDOS (yellow-color) for Zn atoms near divacancies (V_N-V_N) are shown in (a) the (0)-charge state and (b) the (2+)-charge state, from first-principles HSE calculations.

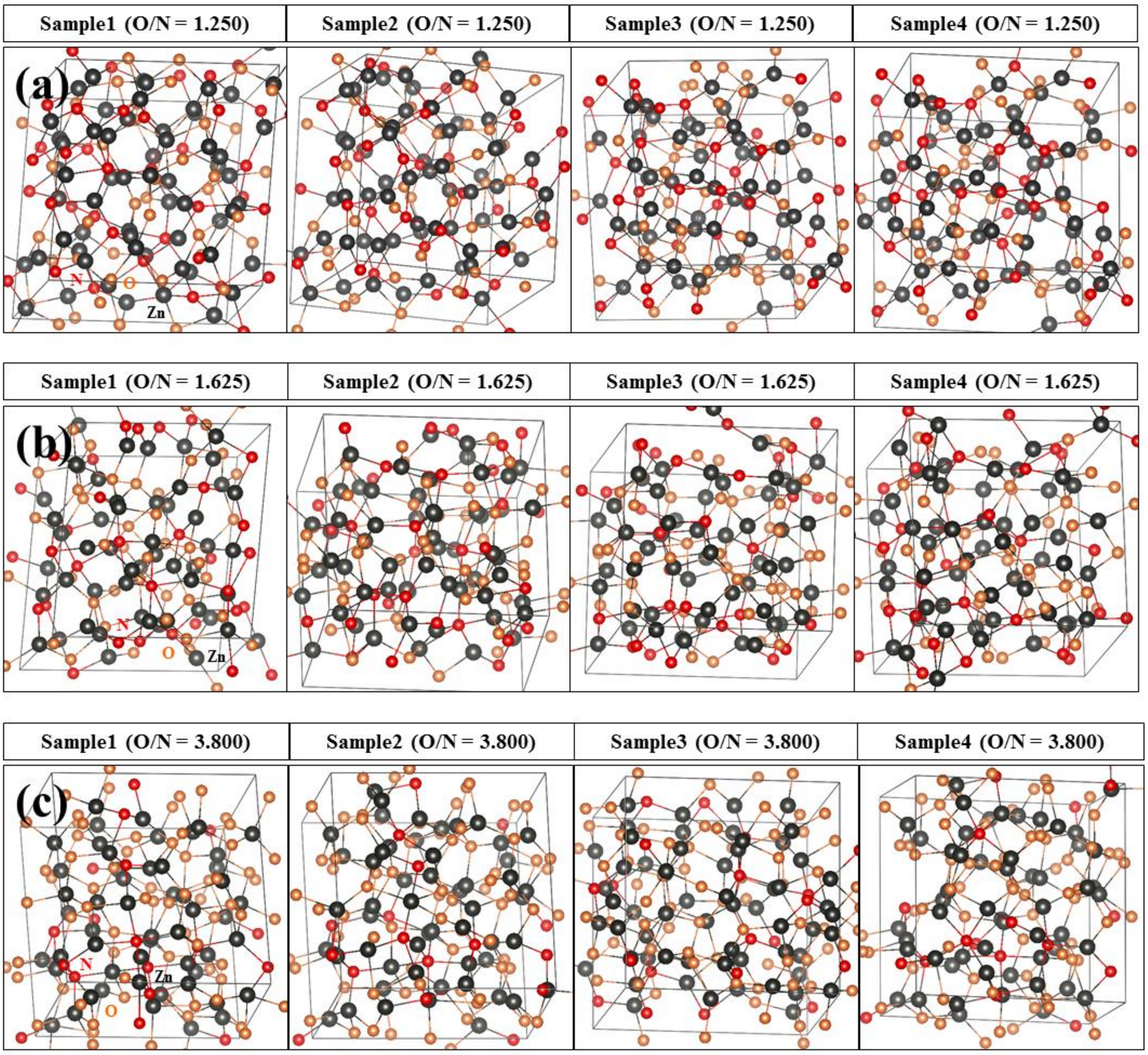

Figure S4. The four atomic structures of stoichiometric amorphous ZnON supercells (generated by melt-and-quench MD simulations) are illustrated for (a) O/N = 1.250, (b) O/N = 1.625, and (c) O/N = 3.800. Here, the gray, orange, and red circles represent Zn, O, and N atoms, respectively.

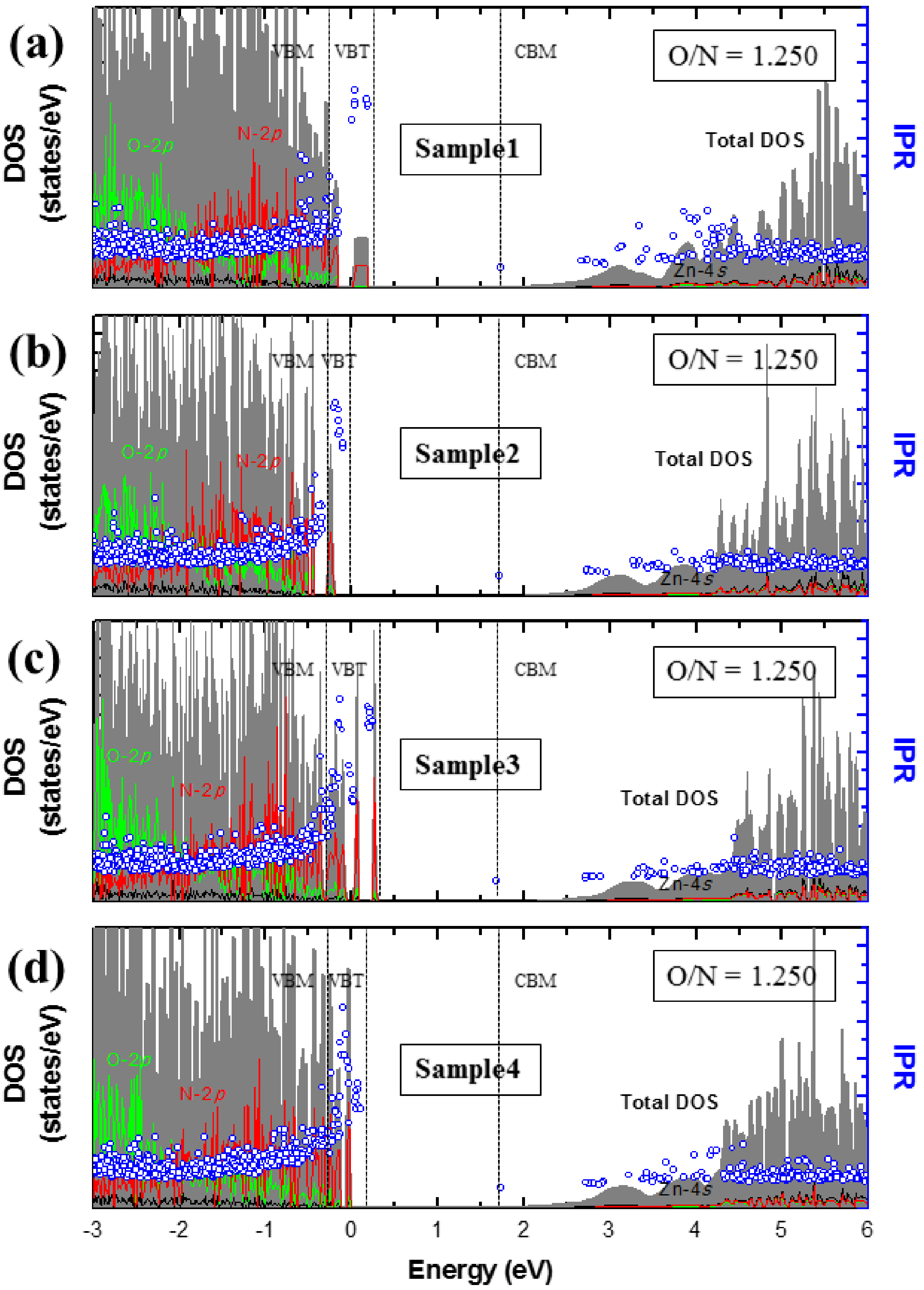

Figure S5. For four stoichiometric amorphous ZnON samples from (a) to (d) with O/N=1.250 anion ratio, the total density of states (DOS), inverse participation ratio (IPR), and PDOS of O-2*p*, N-2*p*, and Zn-4*s* are shown by first-principles HSE calculations.

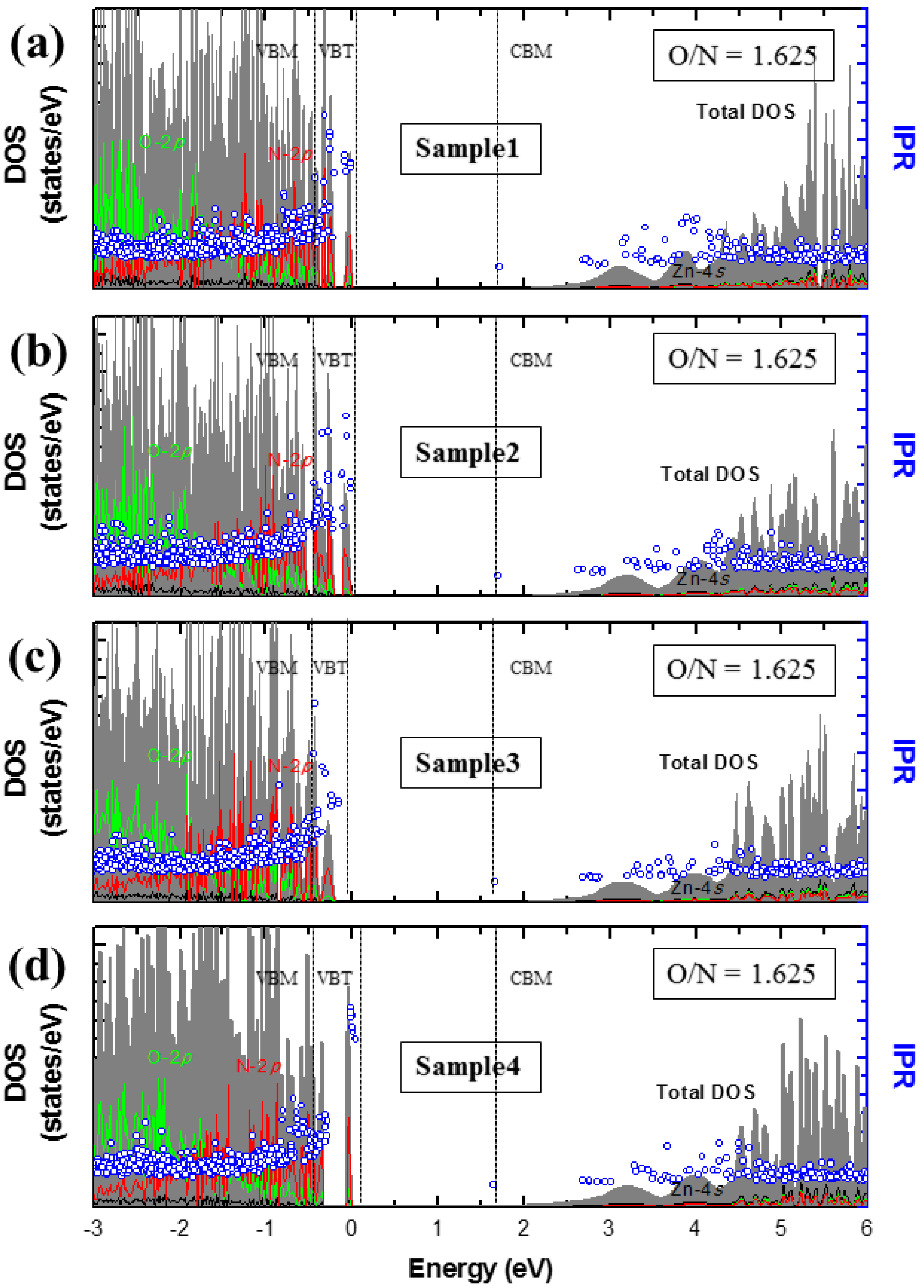

Figure S6. For four stoichiometric amorphous ZnON samples from (a) to (d) with O/N=1.625 anion ratio, the total DOS, IPR, and the PDOS of O-2*p*, N-2*p*, and Zn-4*s* are shown by first-principles HSE calculations.

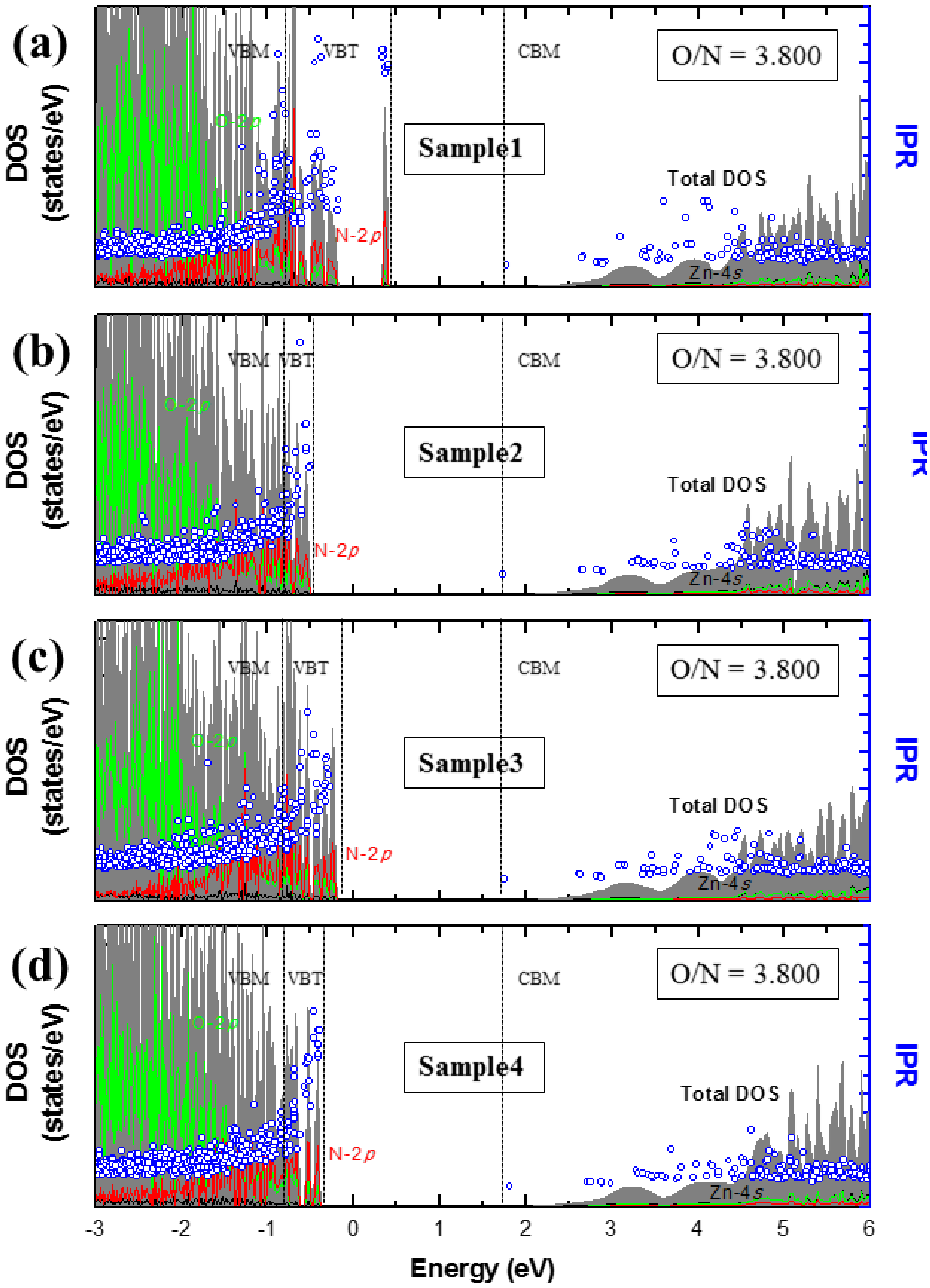

Figure S7. For four stoichiometric amorphous ZnON samples from (a) to (d) with O/N=3.800 anion ratio, the total DOS, IPR, and the PDOS of O-2*p*, N-2*p*, and Zn-4*s* are shown by first-principles HSE calculations.
